# Supplementary material for: DNA replication timing reveals genome-wide features of transcription and fragility
Source: Nat Commun. 2025 May 19;16:4658. doi: 10.1038/s41467-025-59991-w (PMC12089344; doi:10.1038/s41467-025-59991-w)
Supplement: Supplementary file 1 — Supplementary Information [file 41467_2025_59991_MOESM1_ESM.pdf]

Supplementary Information

Supplementary Note 1: Mathematical notes

1.1 Expected time of replication

Without loss of generality, we assume a ring network (periodic DNA) to enforce symmetry of replication with respect to a focal origin. In a large genome, this periodic assumption has minimal influence across most regions, apart from the chromosome ends.

Let T be the time a site takes to either fire (if it is a replication origin) or be replicated by an incoming fork. We can think of T as an explicit function of the origin firing times A_i, where $A_i \overset{\text{iid}}{\sim} \text{Exp}(f)$. In particular, $\mathbb{E}[A_i] = 1/f$. We index each site by its distance from the origin of interest, given by $|i|$. Notice that $i = 0$ corresponds to the focal origin, and v is interpreted as the number of replicated sites per time unit. We have

$$T = \min_i \{A_i + |i|/v\} \tag{S1}$$

since it takes time $|i|/v$ for a replication fork initiated at site i to reach the origin of interest. Then,

$$P(T > t) = \prod_i P(A_i > t - |i|/v) = \prod_i \min\{1, \exp(-f(t - |i|/v))\} \tag{S2}$$

since $A_i > 0$ and $A_i \overset{\text{iid}}{\sim} \text{Exp}(f)$. Hence, the expectation of the replication time for any one site is given by

$$\mathbb{E}[T] = \int_0^\infty P(T > t)\, dt = \int_0^\infty \prod_i \min\{1, \exp(-f(t - |i|/v))\}\, dt. \tag{S3}$$

This integral can be partitioned across each interval for which $|i| \leq vt \leq |i + 1|$. Within these intervals, the integrands adopt the form ae^{-bt}, thereby permitting analytical evaluation. A few particular cases include:

- One origin ($n = 1$):

$$\mathbb{E}[T; 1] = \int_0^\infty e^{-ft} dt = \frac{1}{f} \tag{S4}$$

- Two origins ($n = 2$):

$$\mathbb{E}[T; 2] = \int_0^{\frac{1}{v}} e^{-ft} dt + \int_{\frac{1}{v}}^\infty e^{-f(2t-1/v)} dt = \frac{1}{f}\left(1 - \frac{1}{2} e^{-\frac{f}{v}}\right) \tag{S5}$$

- Three origins ($n = 3$):

$$\mathbb{E}[T; 3] = \int_0^{\frac{1}{v}} e^{-ft} dt + \int_{\frac{1}{v}}^\infty e^{-f(3t-2/v)} dt = \frac{1}{f}\left(1 - \frac{2}{3} e^{-\frac{f}{v}}\right) \tag{S6}$$

- Four origins ($n = 4$):

$$\mathbb{E}[T; 4] = \int_0^{\frac{1}{v}} e^{-ft} dt + \int_{\frac{1}{v}}^{\frac{2}{v}} e^{-f(3t-2/v)} dt + \int_{\frac{2}{v}}^\infty e^{-f(4t-4/v)} dt = \frac{1}{f}\left(1 - \frac{1}{12} e^{-4\frac{f}{v}} - \frac{2}{3} e^{-\frac{f}{v}}\right) \tag{S7}$$

where $\mathbb{E}[T;n] \equiv \mathbb{E}[T]$ for each n. In the general case, the result depends on the parity of n. When n is odd, for each k, there are 2 origins at a distance of $k = 1, 2, \ldots, (n-1)/2$ from the origin of interest. Adding up these distances leads to

$$\mathbb{E}[T; n_{\text{odd}}] = \sum_{k=0}^{(n-3)/2} \int_{k/v}^{(k+1)/v} e^{-f((2k+1)t - k(k+1)/v)}\, dt + \int_{(n-1)/(2v)}^{\infty} e^{-f(nt-(n-1)(n+1)/(4v))}\, dt, \quad \text{(S8)}$$

where the last term is just the $k = (n-1)/2$ term of the sum with the upper limit replaced by ∞. Solving the integrals yields

$$\mathbb{E}[T; n_{\text{odd}}] = \frac{1}{f}\left[\sum_{k=0}^{(n-3)/2} \frac{e^{-fk^2/v} - e^{-f(k+1)^2/v}}{2k+1} + \frac{e^{-f(n-1)^2/(4v)}}{n}\right]. \quad \text{(S9)}$$

When n is even, for each k there are 2 origins at a distance of $k = 1, 2, \ldots, (n-2)/2$, and then there is 1 origin at a distance of $n/2$. Again, we add up the distances, each twice, but since there is only one origin at a distance of $n/2$, the very last distance sum is $n^2/4$. So, we get

$$\mathbb{E}[T; n_{\text{even}}] = \sum_{k=0}^{(n-2)/2} \int_{k/v}^{(k+1)/v} e^{-f((2k+1)t - k(k+1)/v)} dt + \int_{n/(2v)}^{\infty} e^{-f(nt-n^2/(4v))} dt. \quad \text{(S10)}$$

Solving the integrals yields

$$\mathbb{E}[T; n_{\text{even}}] = \frac{1}{f}\left[\sum_{k=0}^{(n-2)/2} \frac{e^{-fk^2/v} - e^{-f(k+1)^2/v}}{2k+1} + \frac{e^{-fn^2/(4v)}}{n}\right]. \quad \text{(S11)}$$

Using the ceiling function $\lceil \cdot \rceil$ to handle parity, a general expression for each origin, and any n, is

$$\mathbb{E}[T; n] \equiv \frac{1}{f}\left[\sum_{k=0}^{\lceil (n-3)/2 \rceil} \frac{e^{-fk^2/v} - e^{-f(k+1)^2/v}}{2k+1} + \frac{e^{-f(\lceil (n-1)/2 \rceil)^2/v}}{n}\right]. \quad \text{(S12)}$$

In particular,

$$\mathbb{E}[T; \infty] \equiv \lim_{n\to\infty} \mathbb{E}[T; n] = \frac{1}{f}\sum_{k=0}^{\infty} \frac{e^{-fk^2/v} - e^{-f(k+1)^2/v}}{2k+1} \quad \text{(S13)}$$

which is Equation (5). Equation (1) arises from a similar reasoning, achieved by expressing the product of exponentials as a single exponential of sums. Although the series $\mathbb{E}[T; n]$ converges for $f > 0$, its closed-form expression is not known. If we rescale time $\tilde{T} \equiv fT$, $\tilde{t} \equiv ft$, and define $x \equiv f/v$, we may rewrite Equation (S12) in a more compact, non-dimensional form

$$\mathbb{E}[\tilde{T}; n] \equiv \sum_{k=0}^{\lceil (n-3)/2 \rceil} \frac{e^{-xk^2} - e^{-x(k+1)^2}}{2k+1} + \frac{e^{-x(\lceil (n-1)/2 \rceil)^2}}{n}. \quad \text{(S14)}$$

As $n \to \infty$, we have

$$\mathbb{E}[\tilde{T}; \infty] \equiv \lim_{n\to\infty} \mathbb{E}[\tilde{T}; n] = \sum_{k=0}^{\infty} \frac{e^{-xk^2} - e^{-x(k+1)^2}}{2k+1} = \sum_{k\in\mathbb{Z}} \frac{e^{-xk^2}}{1-4k^2}. \quad \text{(S15)}$$

A few interesting observations can be made regarding the upper bounds of this limit.

1.2 On Dawson function estimates

The series $g(x) \equiv \mathbb{E}[\tilde{T};\infty]$ is related to the family of theta functions[1], allowing us to express it in terms of

$$\vartheta(x) = \sum_{k\in\mathbb{Z}} e^{-\pi(xk)^2} \tag{S16}$$

which satisfies $\vartheta(1/x) = x\vartheta(x)$. From Equation (S15), g satisfies

$$g(x) + 4g'(x) = \sum_{k\in\mathbb{Z}} e^{-xk^2} = \vartheta(\sqrt{x/\pi}), \tag{S17}$$

and thus

$$g(x) = e^{-x/4}\int_0^{x/4} e^y\,\vartheta(2\sqrt{y/\pi})\,dy. \tag{S18}$$

In particular, for small x we have

$$g(x) = \sqrt{\pi}D_+(\sqrt{x}/2) + O(xe^{-\pi^2/x}) \tag{S19}$$

where

$$D_+(z) = e^{-z^2}\int_0^z e^{t^2}\,dt = \frac{1}{2}\sum_{n=0}^{\infty}\frac{(-1)^n n!}{(2n+1)!}(2z)^{2n+1} \tag{S20}$$

is the Dawson function[2]. A less accurate estimate is then $g(x) = \sqrt{\pi x}/2 + O(x^{3/2})$. Various upper bounds may also be obtained this way. Reverting the change of variables, we get

$$\mathbb{E}[T;\infty] \simeq \frac{1}{2}\sqrt{\frac{\pi}{fv}} \tag{S21}$$

as in Equation (6).

Supplementary Note 2: Computational methods and data

2.1 Beacon Calculus model

As discussed in Boemo et al.[3], a minimal replication model in `bcs` is built around three core processes: replication origins (`ORI`), left-moving forks (`FL`), and right-moving forks (`FR`). These processes are positioned along a chromosome of length `L`, where each process has a unique integer parameter, `i`, representing its specific location between 1 and `L`. In addition, the origins have a replication initiation rate, `fire` (or f in our model), which can be extended to include licensing probabilities in more advanced setups. To track which positions have been replicated, `bcs` uses markers called beacons. Whenever a fork replicates position `i`, it dispatches a beacon on the `chr` channel with the parameter `i`. This beacon indicates that replication is complete at that coordinate, ensuring the model can monitor progress across the entire chromosome.

The following is an example of the `bcs` script with 10 replication origins equally spaced over 100 sites

```
// DNA Replication

// Variables
// Chromosome length
L = 100;
// Fast rate
fast = 100000;
// Fork velocity
v = 1.4;

// Process definitions
ORI[i,fire] = {~chr?[i],fire}.(FL[i]||FR[i]);

FR[i] = {chr![i],fast}.[i < L] -> {~chr?[i+1],v}.FR[i+1];
FL[i] = {chr![i],fast}.[i > 0] -> {~chr?[i-1],v}.FL[i-1];

// Process initiation
ORI[1,0.06048832790213383] || ORI[12,0.002045183033099289]
 || ORI[23,0.0012753405213046796] || ORI[34,0.0011945930278953077]
 || ORI[45,0.001035526093646997] || ORI[56,0.0011165358858784408]
 || ORI[67,0.002560893635329413] || ORI[78,0.003411336829553979]
 || ORI[89,0.0022730688407988954] || ORI[100,0.0038028859830789045];

// End
```

A periodic version of DNA replication can be achieved by changing both `FR` and `FL` process definitions to

```
FR[i] = {chr![i],fast}.(([i<L] -> {~chr?[i+1],v}.FR[i+1]) || ([i==L] -> {~chr?[0],v}.FR[0]));
FL[i] = {chr![i],fast}.(([i>0] -> {~chr?[i-1],v}.FL[i-1]) || ([i==0] -> {~chr?[L],v}.FL[L]));
```

2.2 Fitting the model

2.2.1 Main algorithm

The following code presents the main fitting function, `fitfunction`, used in the fitting algorithm described in the main text. It provides an efficient way of computing Equation (1) to mimic `bcs` simulations for non-uniform firing rates. `fitfunction` accepts four arguments: `list` (a data vector with the RT profile of the entire genome), `v0` (average fork speed, usually set to 1.4 kb/min), and `st0` (radius of influence R, as dedfined in the main text). The first guess `x00` is then constructed based on `list`, by Equation (7). We use an adapted version of `np.roll()`. Data was processed via the Python extension pyBigWig[4]. See https://github.com/fberkemeier/DNA_replication_model.git for further details.

```
# Import dependencies
import cProfile
import math
from time import monotonic
from typing import Any
import numpy as np

# Main function
def fitfunction(list, v0, st0):

        timel = list
        v = v0
        st = st0
        exp_v = np.exp(-1/v)
        x00 = np.array([(math.pi/(4*v))*i**(-2) for i in timel])

        # VECTORIZED APPROACH

        def fast_roll_add(dst, src, shift):
            dst[shift:] += src[:-shift]
            dst[:shift] += src[-shift:]

        def fp(x, L, v):
            n = len(x)
            y = np.zeros(n)
            last_exp_2_raw = np.zeros(n)
            last_exp_2 = np.ones(n)
            unitary = x.copy()
            for k in range(L+1):
                if k != 0:
                    fast_roll_add(unitary, x, k)
                    fast_roll_add(unitary, x, -k)
                exp_1_raw = last_exp_2_raw
                exp_1 = last_exp_2
                exp_2_raw = exp_1_raw + unitary / v
                exp_2 = np.exp(-exp_2_raw)

                # Compute the weighted sum for each j and add to the total
                y += (exp_1 - exp_2) / unitary

                last_exp_2_raw = exp_2_raw
                last_exp_2 = exp_2
            return y

        def fitf(time, lst, x0, j):
                return x0[j] * (lst[j]/time[j])**(2)

        def cfit(time, lst, x0):
            result = np.empty_like(x0)
```

```
        for j in range(len(x0)):
            if fitf(time, lst, x0, j) < 10**(-20):
                result[j] = 10**(-20)
            elif abs(time[j] - lst[j]) < .5:
                result[j] = x0[j]
            else:
                result[j] = fitf(time, lst, x0, j)
        return result

    xs = x00
    my_list = [':.20f'.format(i) for i in xs]
    return my_list
```

2.2.2 Effects of fork speed on replication timing misfits

Our model pairs a fixed fork speed with stochastic origin firing, imposing a natural limit on how steeply replication timing can transition from early to late. Even if more origins fire in a region, they can only flatten this slope; they cannot exceed the fork-speed bound. Consequently, any empirical data showing sharper transitions—often due to fork stalling or replication stress—will remain under-fitted (i.e., predicted to replicate too early). While the fitting algorithm may raise firing rates to accommodate steep timing, it does not systematically inflate them; once the constant-speed ceiling is reached, persistent misfits highlight regions where forks slow or stall beyond our model's assumptions.

This is most clearly illustrated by thinking about the timing curve of a single-origin system: there will be a sharp point at the origin and the gradient elsewhere will be determined by the rate of fork movement. Adding additional origins at any firing rate can only decrease the magnitude of the curve's gradient.

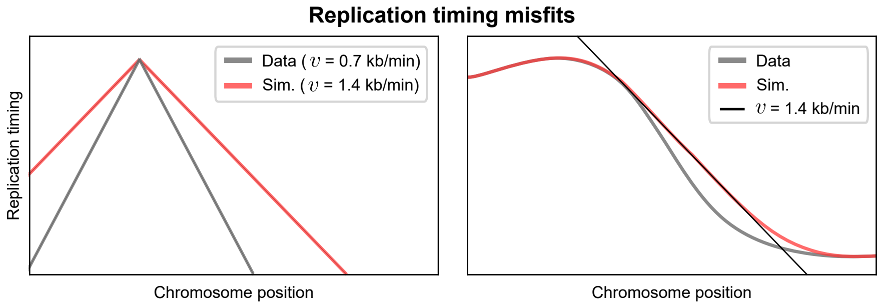

Supplementary Figure 1: Effects of fork speed on replication timing profiles.
Illustration of how a fixed fork speed constrains the steepness of the replication-timing curve. Left: A single-origin "peak" shows that, if the replication fork moves more slowly (gray line, $v = 0.7$ kb/min), the slope is steeper than a faster fixed fork speed can reproduce (red line, $v = 1.4$ kb/min). Allowing extra origins to fire simply flattens slopes rather than raising them above the fork-speed limit. Right: In a multi-origin context, empirical data (gray) can exhibit sharper early-to-late transitions than the model (red) allows. Once fork speed (black diagonal line) is reached, the model cannot replicate any faster, leading to a systematic underestimation of the replication time.

2.3 Data mappability

Repli-seq data often face mappability issues, particularly in regions with repetitive sequences or low complexity, where short DNA reads cannot be accurately mapped[5,6]. Based on data from Hansen et al.[5], these regions of low or problematic mappability account for approximately 20% of the whole genome and around 25% of high-error regions (defined as those with errors exceeding 10^2 min), highlighting their relevance in areas prone to replication timing errors. The mean size of these gaps is approximately 42.37 kb (Supplementary Fig. 2). On average, we observed a phi coefficient of 0.21 when comparing high-error regions and problematic loci, indicating a weak positive correlation between the two. This coefficient, derived from a contingency table, suggests that while there is some overlap between high-error and masked regions, the correlation is not strong. Despite this overlap, mappability issues do not significantly affect overall replication timing analyses, as the majority of high-error regions occur in well-mapped genomic areas, ensuring the reliability of the data. Given the low phi coefficient, we do not exclude these data from our analysis, since the presence of low mappability regions does not appear to be a major factor influencing replication timing errors, allowing us to retain these data in our analysis without compromising its validity.

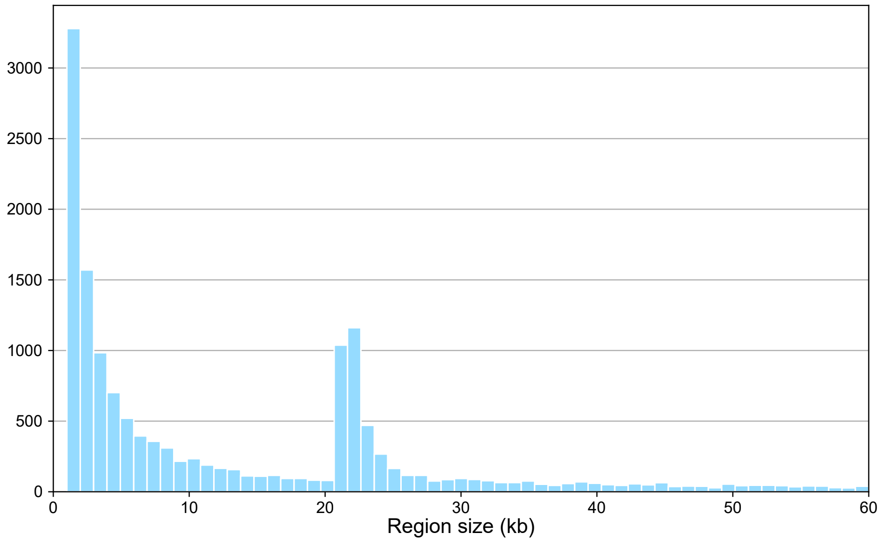

Supplementary Figure 2: Distribution of problematic mappability region sizes.
Histogram showing the distribution of region sizes with low or problematic mappability (in kilobases) across the genome. These regions are excluded from replication timing analyses due to difficulties in accurately mapping sequencing reads. The majority of these regions are small, with peaks around 1-5 kb and another noticeable peak around 20 kb. The mean size of these regions is approximately 42.37 kb.

2.4 Fragility analysis in HCT116

This section expands upon the main text's examination of fragile sites and replication-timing misfits, with a focus on whether specific loci in HCT116 display distinctive error patterns. HCT116 was chosen since confirmatory data on fragile site expression is available[6,7]. The analyses presented here include a high-resolution mapping of misfit regions and additional statistical comparisons of fragile versus non-fragile sites. These efforts help clarify whether fragile sites generally stand out from the rest of the genome in terms of replication timing errors, or if notable differences only arise at certain chromosomes or loci.

2.4.1 Misfit regions in HCT116

We begin by compiling a chromosome-scale overview of replication misfits in HCT116, focusing on regions where high error levels overlap with known fragile sites. Supplementary Fig. 3 aligns misfit values with fragile sites, making it possible to see whether errors concentrate in these loci or appear at similar levels elsewhere in the genome. In some cases, fragile sites show misfits comparable to surrounding regions. In others, they deviate markedly, suggesting that local or cell-line-specific factors may be at play. An accompanying table (supplementary file '`misfit_genes_HCT116.xlsx`') lists each gene found within high-misfit regions, along with coordinates, length, and fragile-site status. This resource helps identify instances where replication-timing anomalies coincide with genes of particular interest, such as large or transcriptionally active loci. By linking these observations to fragile-site positions, we can pinpoint areas that may warrant further study. All downstream analyses exclude centromeres, telomeres, assembly gaps, and bins with low mappability.

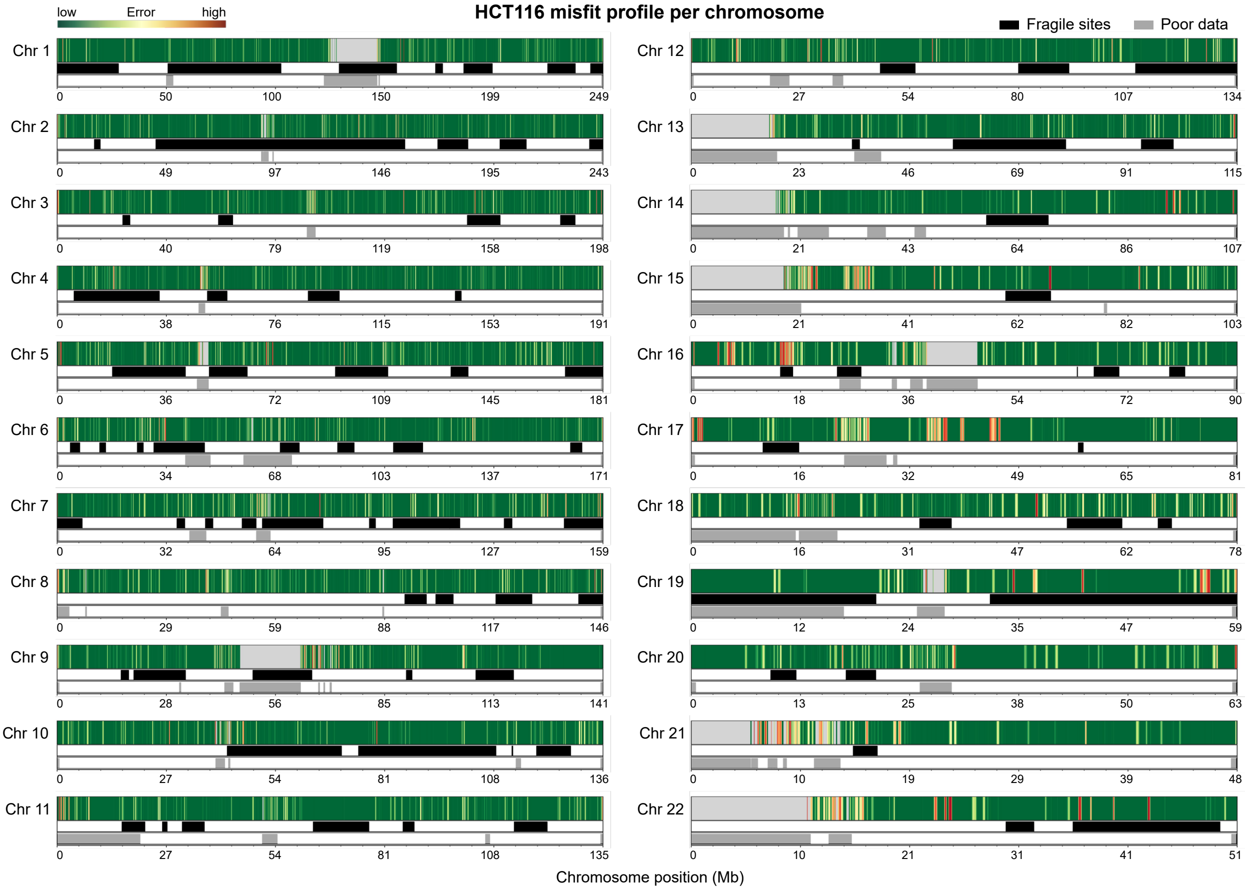

Supplementary Figure 3: Whole-genome misfit profiles of HCT116 cells.
Error heatmaps produced by the main model fitted to Repli-seq data for all chromosomes of HCT116 cells. Black bands indicate fragile sites reported in the HumCFS database, whereas gray bands denote poorly fit segments, including centromeres, telomeres, and regions with known mappability issues.

2.4.2 Statistical analysis

To investigate whether fragile sites (FS) differ from non-fragile sites (nonFS) in replication error distributions, we carry out two complementary comparisons. First, we examine the entire set of genomic positions, comparing all FS with nonFS without applying any error threshold. Second, we introduce a cutoff of 10^2 (min^2) to define high-error positions and compare FS and nonFS solely within this subset. By setting the threshold at 10^2, we capture sufficiently large samples of positions that exceed moderate error values, ensuring both statistical power and biological relevance. This two-tiered approach enables us to assess broad differences in replication error profiles (the full dataset) as well as to focus specifically on positions where replication stress is presumably more pronounced (the high-error subset).

When we consider all genomic positions, FS and nonFS show highly significant differences in both replication error and replication timing. Welch's t-tests, Mann–Whitney U tests, and Kolmogorov–Smirnov tests all return near-zero p-values (Supplementary Fig. 4a), reflecting sig-

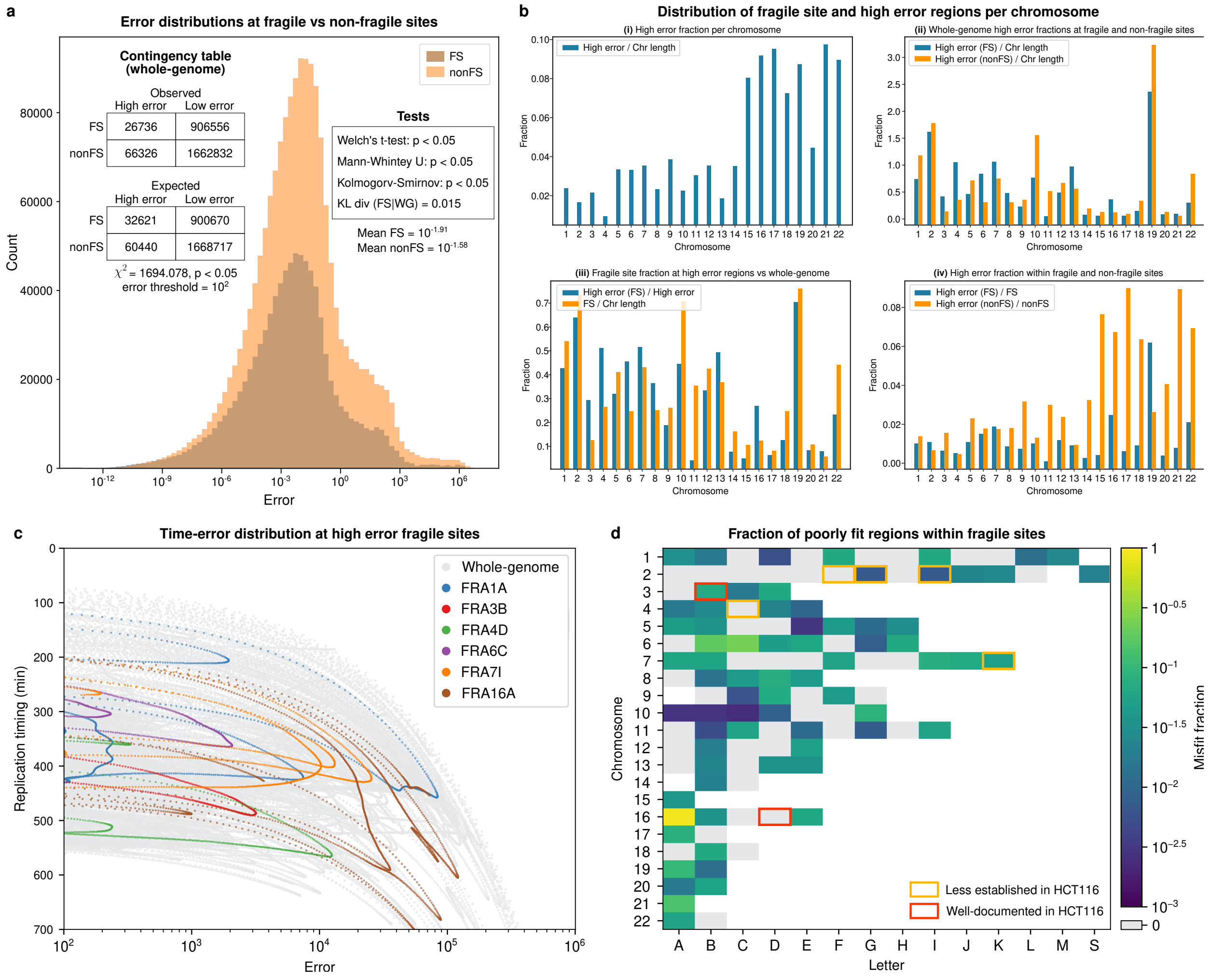

Supplementary Figure 4: Error statistics in HCT116 fragile sites.
a Error distribution on fragile sites (FS) and non-fragile sites (nonFS). Left of histogram: contingency tables for observed and expected counts of FS/nonFS and high/low errors, and Chi-square results, with an error threshold of 10^2. Right: statistical tests results between FS and nonFS error distributions. **b** Overview of how replication errors and fragile sites distribute across multiple comparisons: **(i)** The fraction of high-error loci among all loci on the chromosome, **(ii)** the fraction of high-error loci at FS vs. the fraction of high-error loci at nonFS (both normalized by total chromosome length), **(iii)** the fraction of FS among high-error loci vs. the fraction of FS among all loci, and **(iv)** the fraction of high-error FS among all FS vs. the fraction of high-error nonFS among all nonFS. **c** Relication timing vs. error at poorly fitted fragile sites. **d** Heatmap displaying the high error fractions of fragile sites, across all sites reported in HumCFS. Well-documented (FRA3B and FRA16D) and less established (FRA2F, FRA2G, FRA2I, FRA4C, and FRA7K) FS in HCT116 are highlighted.

nificant shifts in their means, medians, and overall shapes. Large negative t-values indicate that, on average, FS exhibit lower errors and replicate earlier than nonFS. We also compare the one-dimensional error distribution at FS with that of the genome at large using the Kullback–Leibler divergence $D_{\mathrm{KL}} \approx 0.015$, suggesting a moderate departure between these distributions. Extending this analysis to the two-dimensional time-error distribution yields a slightly higher $D_{\mathrm{KL}} \approx 0.03$, confirming that FS differ from the genome in a joint replication context, yet not to an extreme degree.

Focusing on regions with errors above 10^2, we see a strong association between FS status and error classification (Chi-square = 1694.078, $p \approx 0$; contingency table in Supplementary Fig. 4a). Restricting our analysis to these high-error loci, Welch's t-test suggests that the mean error is slightly higher at FS ($t = 3.707$, $p = 2.1 \times 10^{-4}$), while replication timing is earlier ($t = -6.172$, $p = 6.78 \times 10^{-10}$). The two-dimensional Hotelling's T^2 result ($T^2 = 69.748$, $p = 7.77 \times 10^{-16}$) confirms that FS maintain distinct replication features even within this high-error subset. In other words, FS are somewhat under-represented among positions exceeding the threshold, yet those that do exceed 10^2 display a characteristic profile of modestly elevated errors and notably earlier replication timing compared to other high-error regions. These findings also point to potential fragile sites in HCT116 that remain understudied, highlighting them as candidates for closer experimental investigation.

At the genome-wide level, these findings suggest that FS often exhibit fewer extreme errors than would be expected by chance. However, more detailed analyses of specific chromosomes, where FS in HCT116 are well documented[8,6], reveal substantial variability in how these errors manifest across different genomic contexts. Supplementary Fig. 4b shows the chromosome-based distributions of errors and FS vs. nonFS, illustrating the FS-dependent nature of replication-stress patterns. Of particular note is the increasing high-error fraction on chromosomes 3 and 16, and the distinct dynamics on chromosome 19. Supplementary Fig. 4c highlights high-error time–error distributions of poorly fit FS, while Supplementary Fig. 4d presents the overall high-error fraction in FS from the HumCFS database[9]. Interestingly, our model flags the rare fragile site FRA16A as especially problematic[10,11] and predicts replication-stress signatures at the well-established FRA3B[12,13]. In contrast, the model appears to fit well at FRA16D, which aligns with studies challenging its instability in HCT116[7]. Our analysis also identifies less established FS in HCT116—FRA2G, FRA2I, and FRA7K[7,14,15]—as potentially of interest.

Overall, these findings show that although fragile sites frequently show statistically significant differences in replication misfits, particularly on certain chromosomes, this pattern does not hold uniformly across the entire genome. Further targeted studies may help untangle how cell-line-specific factors shape these localized vulnerability profiles within broader replication error landscapes. Nonetheless, by highlighting potential hotspots of replication stress, our model provides a valuable starting point for deeper experimental investigations into the molecular basis of fragility. All tests and supporting code are provided in the GitHub repository: https://github.com/fberkemeier/DNA_replication_model.git.

2.5 Data correlations

Here, we present a comparison of different statistical tests applied to the datasets discussed in the main text. This analysis evaluates the relationships between replication timing error, firing rates, and transcriptional or chromatin features, providing insights into the suitability and results of Pearson, Spearman rank, and Kendall's tau tests for these data. Pearson, Spearman rank, and Kendall's tau offer distinct advantages based on the nature of the data and relationships analysed. Pearson is suited for continuous, normally distributed data with linear relationships, while Spearman rank excels with non-linear or ordinal data by capturing monotonic trends through ranked values. Kendall's tau is particularly effective for smaller datasets, using concordant and discordant pairs to measure associations. Given the non-linear and ranked nature of replication metrics, Spearman rank is ideal for our analysis. Supplementary Fig. 5 shows the correlations between replication timing error, firing rates, and transcriptional or chromatin features, demonstrating the relevance of these tests to our data.

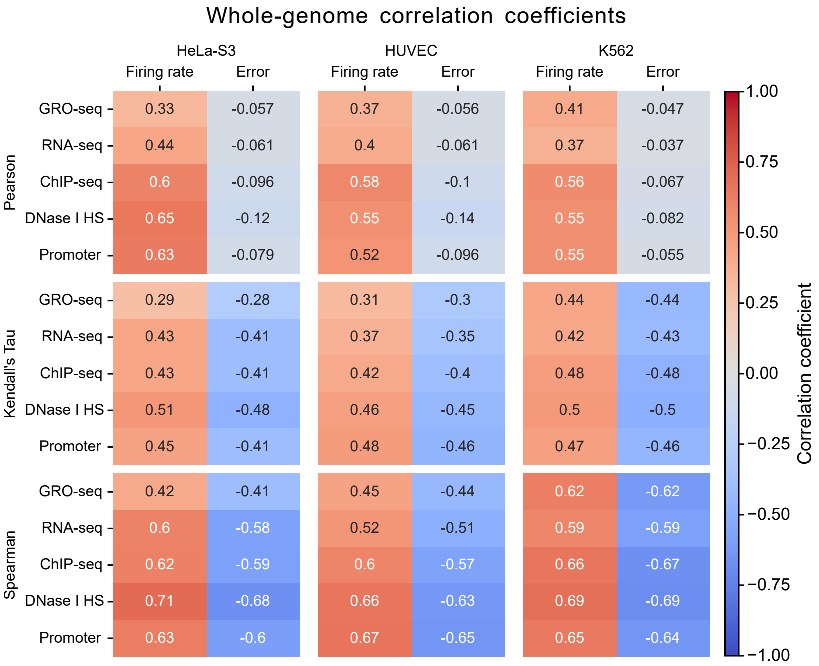

Supplementary Figure 5: Correlations between replication, transcription and chromatin data. Heatmap displaying the Spearman, Kendall's Tau, and Pearson correlation coefficients between origin firing rates and fit errors with transcriptional and chromatin features for HeLa, HUVEC, and K562 cell lines. All tests returned p-value $< 10^{-15}$.

2.6 Theoretical digression: Application to *Saccharomyces cerevisiae*

Although our primary analyses focus on the human genome, the underlying framework is fully generalizable to other eukaryotes. To demonstrate this, we apply our fitting pipeline to *S. cerevisiae* (budding yeast) replication-timing data[16,17], where the most active origins are known[18]. In particular, we test whether our model is able to recover the well-established origins in yeast. To adapt to yeast's shorter genome, we constrain the neighbour-sum in Equation (1) for each site j to

$$\max(1-j,-k) \leq i \leq \min(n-j,k) \tag{S22}$$

instead all $|i| \leq k$. Choosing the radius of influence R to equal each chromosome's length then handles chromosome ends automatically, without altering any core assumptions.

To assess whether our model can recover known replication origins in yeast, we fit firing rates to replication timing data from Müller et al.[16] (Supplementary Fig. 6a), and compare the results to autonomously replicating sequences (ARS) annotations, independently obtained from the OriDB database[18], selecting those origins marked as 'Confirmed' or 'Likely'. This yields a one-dimensional profile of firing rates across the genome, along with a binary indicator vector specifying whether each genomic position falls within an annotated origin interval. We find that firing rates are systematically higher within these intervals (Supplementary Fig. 6b), and that the model recovers > 86 % of the origins at high-firing rates within $\pm$ 2 kb. To quantify this enrichment, we applied a Mann–Whitney U test and a point-biserial correlation to evaluate the association between the binary origin label and the continuous firing rate. These tests produced highly significant results ($p \leq 10^{-12}$), indicating that the model successfully recovers regions of known origin activity.

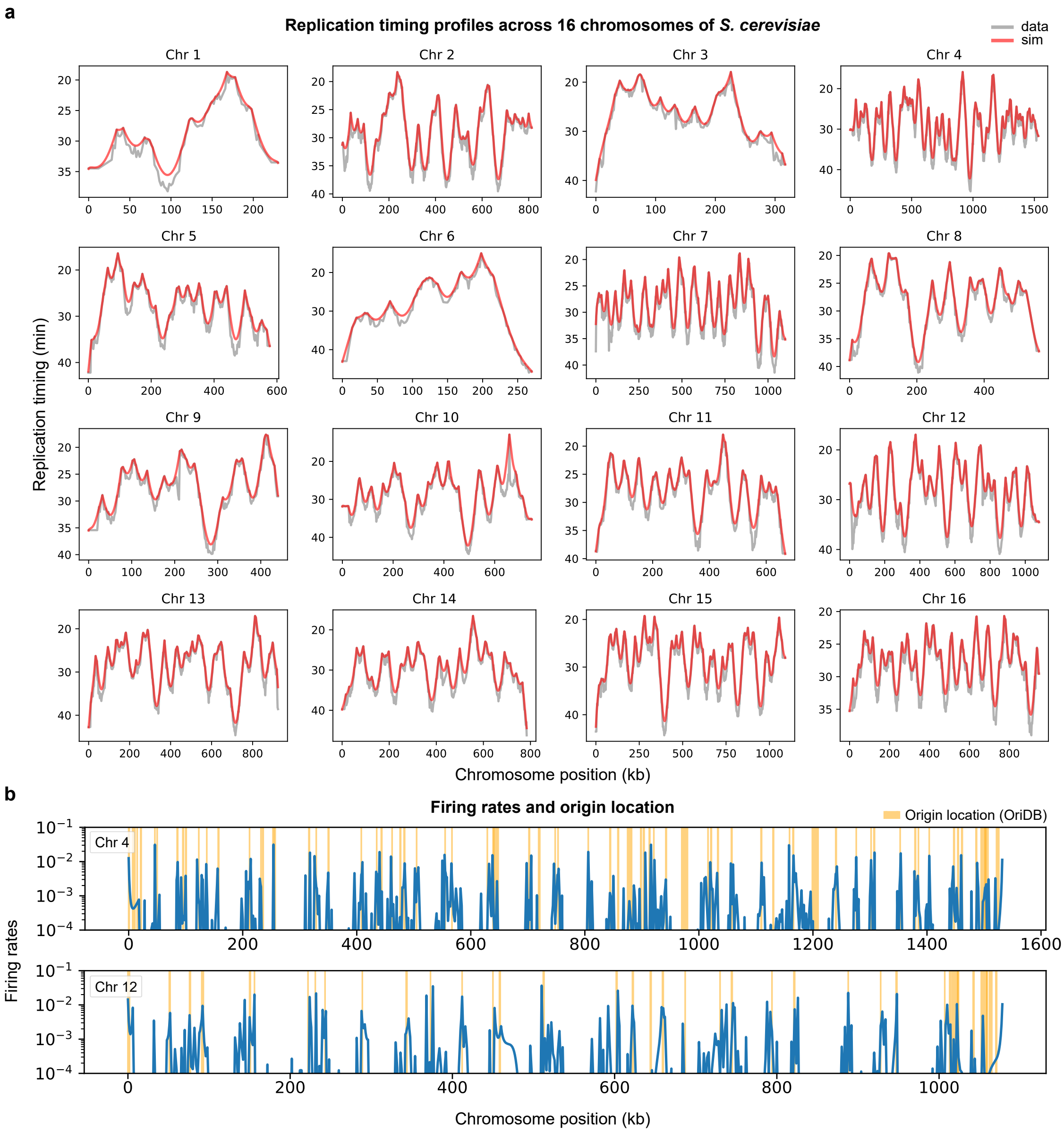

Supplementary Figure 6: Fitting the model in *S. cerevisiae*.
a Observed replication timing from Müller et al.[16] compared with simulated timing profiles across the entire yeast genome, fitted using Equation (1) on each full chromosome. **b** Examples of fitted firing-rate profiles for chromosomes 4 and 12, highlighting sharp peaks at known origin locations from the OriDB database[18] ('Confirmed' and 'Likely'). The model infers these peaks from timing data alone, effectively suppressing firing activity in non-origin regions and recovering known origin locations without prior information.

Supplementary Tables

Supplementary Table 1: Table listing the ten largest genes exhibiting misfits across all chromosomes, ranked from largest to smallest (left to right). Genes located at fragile sites are annotated as follows: C for common fragile sites, R for rare fragile sites, and CR for genes reported at both. All gene annotations refer to H1 cells with Repli-seq data aligned to the hg38 genome.

Chr	Misfit genes across common (C) and rare (R) fragile sites									
1	*AGBL4*[C]	*KAZN*[C]	*NEGR1*[C]	*RABGAP1L*[C]	*RYR2*	*DNM3*	*ST6GALNAC3*[C]	*KCNH1*	*HMCN1*	*PLD5*
2	*LRP1B*[R]	*DPP10*[R]	*NRXN1*[R]	*THSD7B*[R]	*NCKAP5*[R]	*CNTNAP5*[R]	*ALK*	*AFF3*	*MYT1L*[R]	*KCNS3*[C]
3	*FHIT*[C]	*RBMS3*[C]	*TBC1D5*	*ROBO1*	*LSAMP*	*CADM2*	*CACNA2D3*	*EPHA6*[C]	*ZBTB20*	*LPP*
4	*FSTL5*[C]	*LRBA*[C]	*CFAP299*[C]	*RASGEF1B*[C]	*ANK2*	*TENM3*	*SORCS2*	*STK32B*	*MAML3*	*AFG2A*
5	*PDE4D*[C]	*TENM2*	*CDH18*[C]	*SGCD*	*SLIT3*	*SPOCK1*	*FER*	*EDIL3*[C]	*HCN1*	*FBXL7*
6	*PRKN*[C]	*NKAIN2*[C]	*GRIK2*	*ADGRB3*	*GMDS*	*FARS2*	*PKHD1*[C]	*TRDN*	*SLC35F1*	*ZDHHC14*
7	*CNTNAP2*[C]	*MAGI2*[C]	*DPP6*[C]	*SDK1*[C]	*IMMP2L*	*DGKB*	*SUGCT*	*BBS9*	*CDK14*	*ELMO1*[C]
8	*NRG1*	*VPS13B*	*NKAIN3*	*UNC5D*	*XKR4*	*EXT1*[C]	*MCPH1*	*ASPH*	*EBF2*	
9	*PTPRD*	*LINGO2*	*ADAMTSL1*	*TRPM3*	*DENND1A*	*BNC2*	*ROR2*[C]	*NFIB*	*RFX3*	*SLC24A2*
10	*PCDH15*[R]	*NRG3*[R]	*KCNMA1*[R]	*GRID1*[R]	*PARD3*	*ANK3*[C]	*SORCS1*	*PLXDC2*	*CACNB2*	*ABLIM1*
11	*DLG2*[C]	*LRRC4C*[C]	*CNTN5*	*NELL1*[CR]	*TENM4*	*NAV2*[CR]	*KIRREL3*	*GRM5*	*SOX6*[CR]	*DCDC1*
12	*ANKS1B*[C]	*MGAT4C*[C]	*TMTC2*[C]	*ANO4*	*TRHDE*	*CNTN1*	*SLC2A13*	*ABTB3*	*PTPRO*[R]	*SLCO1B3-B7*
13	*NBEA*[C]	*MYO16*	*KLHL1*	*DCLK1*	*CLYBL*	*FREM2*	*CLDN10*	*CAB39L*	*SCEL*	*TNFRSF19*
14	*RAD51B*[C]	*GPHN*	*TTC6*	*TSHR*	*TRAF3*	*CDC42BPB*	*BAZ1A*	*MIA2*	*LIN52*	*SOS2*
15	*UNC13C*	*FMN1*	*ADAMTS17*	*IGF1R*	*APBA2*	*LRRC49*	*RNF111*	*RFX7*	*SHC4*	*TRPM7*
16	*WWOX*[C]	*RBFOX1*[C]	*CDH13*	*GSE1*	*FTO*	*ZNF423*	*ITFG1*	*ACSM3*	*ADAMTS18*	*CFDP1*
17	*ASIC2*	*SHISA6*	*ACACA*	*SPECC1*	*ARSG*	*VMP1*	*MAP2K4*[R]	*SMURF2*	*TADA2A*	*DHRS7B*
18	*DCC*	*DLGAP1*	*CCDC178*[C]	*L3MBTL4*	*LDLRAD4*	*KIAA1328*	*NEDD4L*[C]	*LOXHD1*	*MAPK4*[C]	*CCDC102B*
19	*MARK4*[C]	*INSR*	*MUC16*	*TDRD12*	*ZNF83*	*URI1*	*ZNF569*[C]	*NLRP11*	*AP1M1*	*NLRP4*[C]
20	*PTPRT*	*PLCB1*[C]	*PLCB4*	*PAK5*	*EYA2*	*RIN2*	*SYNDIG1*	*NCOA3*	*ZFP64*	*RALGAPB*
21	*CHODL*	*GET1-SH3BGR*	*TTC3*	*SH3BGR*						
22	*BCR*	*DGCR2*	*GAB4*	*YPEL1*	*PPIL2*	*MAPK8IP2*	*ARSA*			

Note: Gene names are presented in italics following conventional nomenclature.

References

1. Andrey N. Tyurin. Quantization, classical and quantum field theory and theta-functions. Preprint at https://arxiv.org/abs/math/0210466 (2002).

2. Nico M. Temme. *Error Functions, Dawson's and Fresnel Integrals* (NIST, Gaithersburg, 2010).

3. Michael A. Boemo, Luca Cardelli & Conrad A. Nieduszynski. The beacon calculus: a formal method for the flexible and concise modelling of biological systems. *PLOS Computational Biology* **16**, e1007651 (2020).

4. Devon Ryan *et al.* pyBigWig. Zenodo, https://doi.org/10.5281/zenodo.5144144 (2021).

5. R. Scott Hansen *et al.* Sequencing newly replicated DNA reveals widespread plasticity in human replication timing. *Proceedings of the National Academy of Sciences* **107**, 139–144 (2010).

6. Peiyao A. Zhao, Takayo Sasaki & David M. Gilbert. High-resolution Repli-seq defines the temporal choreography of initiation, elongation and termination of replication in mammalian cells. *Genome Biology* **21**, 1–20 (2020).

7. Lora Boteva *et al.* Common fragile sites are characterized by faulty condensin loading after replication stress. *Cell Reports* **32**, 108189 (2020).

8. S. G. Durkin, M. F. Arlt, N. G. Howlett & T. W. Glover. Depletion of CHK1, but not CHK2, induces chromosomal instability and breaks at common fragile sites. *Oncogene* **25**, 4381–4388 (2006).

9. Rajesh Kumar *et al.* HumCFS: a database of fragile sites in human chromosomes. *BMC Genomics* **19**, 985 (2019).

10. Grant R. Sutherland. Rare fragile sites. *Cytogenetic and Genome Research* **100**, 77–84 (2003).

11. J. K. Nancarrow *et al.* Implications of FRA16A structure for the mechanism of chromosomal fragile-site genesis. *Science* **264**, 1938–1941 (1994).

12. Seyed A. Hosseini *et al.* Common chromosome fragile sites in human and murine epithelial cells and FHIT/FRA3B loss-induced global genome instability. *Genes, Chromosomes and Cancer* **52**, 1017–1029 (2013).

13. Patrizia Vernole *et al.* Common fragile sites in colon cancer cell lines: role of mismatch repair, RAD51 and poly(ADP-ribose) polymerase-1. *Mutation Research / Fundamental and Molecular Mechanisms of Mutagenesis* **712**, 40–48 (2011).

14. Zaira M. Limongi, Angela Curatolo, Franca Pelliccia & Angela Rocchi. Biallelic deletion and loss-of-expression analysis of genes at FRA2G common fragile site in tumour-derived cell lines. *Cancer Genetics and Cytogenetics* **161**, 181–186 (2005).

15. Audesh Bhat, Parker L. Andersen, Zhoushuai Qin & Wei Xiao. REV3, the catalytic subunit of Pol ζ, is required for maintaining fragile-site stability in human cells. *Nucleic Acids Research* **41**, 2328–2339 (2013).

16. Carolin A. Müller *et al.* The dynamics of genome replication using deep sequencing. *Nucleic Acids Research* **42**, e3 (2014).

17. Rosie Berners-Lee, Eamonn Gilmore, Francisco Berkemeier & Michael A. Boemo. Regulation of replication timing in *Saccharomyces cerevisiae.* Preprint at https://doi.org/10.1101/2024.10.000000 (2024).

18. Cheuk C. Siow, Sian R. Nieduszynska, Carolin A. Müller & Conrad A. Nieduszynski. OriDB, the DNA replication origin database updated and extended. *Nucleic Acids Research* **40**, D682–D686 (2012).
